# Abnormal Glucose Tolerance in Women Diagnosed With Gestational Diabetes (WHO 2013) 10 Years After Index Pregnancy

**DOI:** 10.1210/jendso/bvae013

**Published:** 2024-01-30

**Authors:** Oratile Kgosidialwa, Christine Newman, Louise Carmody, Brian McGrath, Paula M O’Shea, Fidelma Dunne

**Affiliations:** College of Medicine, Nursing and Health Sciences, National University of Ireland Galway, Galway, H91TK33, Ireland; College of Medicine, Nursing and Health Sciences, National University of Ireland Galway, Galway, H91TK33, Ireland; College of Medicine, Nursing and Health Sciences, National University of Ireland Galway, Galway, H91TK33, Ireland; College of Medicine, Nursing and Health Sciences, National University of Ireland Galway, Galway, H91TK33, Ireland; College of Medicine, Nursing and Health Sciences, National University of Ireland Galway, Galway, H91TK33, Ireland; College of Medicine, Nursing and Health Sciences, National University of Ireland Galway, Galway, H91TK33, Ireland

**Keywords:** gestational diabetes, abnormal glucose tolerance, cardiovascular risk factors

## Abstract

**Context:**

It is not clear if the risk of abnormal glucose tolerance (AGT) is attenuated in the long-term in women diagnosed with gestational diabetes (GDM) using the World Health Organization (WHO) 2013 criteria and who have received appropriate treatment during pregnancy.

**Objective:**

We aimed to assess the long-term prevalence of AGT and other cardiovascular disease (CVD) risk factors in this cohort.

**Methods:**

A retrospective cohort follow-up study was conducted of 37 and 107 women diagnosed with and without GDM respectively using the WHO 2013 criteria between June 2010 and December 2010. Women were invited to attend our center, where they underwent a 75-g oral glucose tolerance test, blood and urine collection, body measurements, and electrocardiography. Main outcome measure included the development of AGT using the American Diabetes Association criteria.

**Results:**

Sixteen (43.2%) women with GDM compared to 5 (4.7%) women with normal glucose tolerance (NGT) at index pregnancy had AGT (*P* < .001). In the GDM group, 10 (27.0%), 7 (18.9%), and 4 (10.8%) women had impaired fasting glucose (IFG), impaired glucose tolerance (IGT), and type 2 diabetes mellitus (T2DM), respectively. In the NGT group, 2 (1.9%), 3 (2.8%), and 1 (0.9%) woman had IFG, IGT, and T2DM, respectively. Women with AGT also had an unfavorable metabolic profile including obesity, hypertension, insulin resistance, and dyslipidemia.

**Conclusion:**

Women treated for GDM (WHO 2013 criteria) remain at increased risk for developing AGT and adverse CVD risk factors as early as a decade after diagnosis. Continued efforts are needed to accurately follow this population to address modifiable risk factors.

Gestational diabetes mellitus (GDM), a common metabolic condition complicating pregnancy, is associated with adverse short and long-term outcomes both for mother and child [1-3]. The first diagnostic criteria for GDM (1960s) were developed to identify women at increased risk of developing type 2 diabetes mellitus (T2DM) later in life [4]. After multiple modifications to the diagnostic criteria for GDM [5, 6], the International Association of Diabetes and Pregnancy Study Groups (IADPSG) criteria were introduced in 2010. It was hoped that this would standardize and harmonize the diagnosis of GDM [7]. These criteria were primarily based on evidence from the Hyperglycemia and Adverse Pregnancy Outcome (HAPO) trial but also included evidence from prior cohorts and systematic reviews that showed a positive and continuous correlation between maternal glucose levels below those recommended at the time and poor perinatal outcomes [1, 7]. These criteria were subsequently adopted by the World Health Organization (WHO) in 2013.

Women with GDM have an increased risk of developing T2DM [8] and premature cardiovascular disease (CVD) in later life, especially in the setting of a positive family history of diabetes [9]. Offspring of women with GDM are at an increased risk of glycemic perturbations themselves evident as young as childhood [2].

Evidence is consistent that regardless of diagnostic criteria used to diagnose GDM, this cohort of women remains at increased risk of developing abnormal glucose tolerance (AGT) in later life compared to women with normal glucose tolerance (NGT) during pregnancy [10]. Women diagnosed with GDM using the WHO 2013 criteria, which uses lower glucose cutoff points, also show increased risk of developing AGT. However, most of the long-term studies analyzed retrospectively applied the WHO 2013 criteria to women who may not have received treatment during pregnancy [11]. Thus, evidence on the long-term maternal metabolic health of women diagnosed and treated for prior GDM using the WHO 2013 criteria in routine clinical practice is lacking. This study sought to assess the long-term prevalence of AGT and adverse CVD risk factors after a diagnosis and treatment of GDM using the WHO 2013 criteria.

## Materials and Methods

### Ethics Approval

Ethical approval for this retrospective cohort study was granted by the Galway Clinical Research Committee (Ref C.A 2159).

### Setting and Participants

Women who had a 75-g oral glucose tolerance test (OGTT) between June and December 2010 were identified through the ILAB laboratory information system and through the ATLANTIC DIP (http://atlanticdipireland.com/) database. The ATLANTIC DIP is an initiative developed to improve health care outcomes of women with diabetes in pregnancy along the Irish Atlantic seaboard through research and collaboration. Universal screening for GDM was applied during this time. Women were diagnosed with GDM according to the WHO 2013 criteria on OGTT if they satisfied one or more of the following: fasting, 1-hour or 2-hour glucose of more than or equal to 5.1 (91.8 mg/dL), 10 (180.0 mg/dL), or 8.5 (153 mg/dL) mmol/L, respectively, after an overnight fast. Clinical details of women with prior GDM were extracted from the DIAMOND diabetes management software system (https://www.hicom.co.uk/diamond) and paper medical records. Clinical details of women with NGT during pregnancy were extracted from paper medical records.

Women excluded from our study were those who were unable to provide informed consent, with pre-GDM at index pregnancy, diagnosed with GDM using other non-WHO 2013 criteria or with an unclear diagnosis (eg, women who fit the WHO 2013 diagnostic criteria but were not offered treatment). Women satisfying the inclusion criteria were invited to participate via post, with a follow-up phone call for those who had not made contact. Initially we had intended to recruit all women from our center and the other 4 ATLANTIC DIP sites but further recruitment was curtailed by the COVID-19 pandemic. All eligible women with prior GDM at index pregnancy were invited to participate. Eligible women with NGT at index pregnancy were invited in batches of 100 women selected randomly from one center. Although we had originally planned to invite all women with NGT, only two 100-women batches were invited due to the COVID-19 pandemic.

### Study Visit

Following informed written informed consent, women attended a 1-day visit at our clinical research facility for an OGTT, blood and urine tests, body measurements, and cardiac electrocardiogram (ECG). Participants were encouraged to continue their normal diet in the days leading up to the visit. Whole blood was collected from fasting women for glucose, insulin, c-peptide, lipids, glycated hemoglobin A_1c_ (HbA_1c_), and other routine biochemistry prior to oral glucose administration. A 75-g glucose load (300 mL of Rapilose) was administered after at least 8 hours of an overnight fast. Because the half-life of metformin is 17.6 hours (https://www.accessdata.fda.gov), patients with a known history of T2DM on metformin were asked to hold their medications for 5 days prior to attendance to enable measurements of their glucose parameters. These women were informed to monitor their blood glucose levels closely during this time. Blood pressure was measured twice at least 1 hour apart using a calibrated automated blood pressure monitor (Mindray, VS-900). Weight and height were measured using the SECA 769 clinical scale without shoes. Waist and hip circumference measurements were made according to the WHO guidelines [12]. A history of CVD was self-reported. All ECGs were reviewed by a cardiologist.

### Primary and Secondary Outcomes

The primary outcome was development of AGT defined as prediabetes or T2DM according to the American Diabetes Association criteria and similar to the HAPO follow-up study (HAPO-FUS) [13] to allow for comparison of prevalence. Prediabetes was defined as fasting plasma glucose (FPG) between 5.6 and 6.9 mmol/L (100-125 mg/dL) and a 2-hour plasma glucose (PG) between 7.8 and 11.0 mmol/L (140-199 mg/dL), or both, during the OGTT. T2DM was self-reported or defined as an FPG of 7.0 mmol/L (126 mg/dL) or more, a 2-hour PG of 11.1 mmol/L (200 mg/dL) or more, or both, during the OGTT.

Secondary outcomes included CVD and CVD risk factors. CVD was defined as self-reported myocardial infarction, heart failure, stroke, or peripheral vascular disease. CVD risk factors documented included body mass index (BMI), waist-to-hip ratio, blood pressure, lipid profile, and insulin resistance. Insulin resistance was estimated using the homeostatic model assessment for insulin resistance (HOMA-IR) ((fasting insulin (μIU/mL) × fasting glucose (mmol/L))/22.5) model. Metabolic syndrome was defined as any 3 of waist circumference greater than or equal to 88 cm, triglycerides greater than or equal to 1.7 mmol/L (or on treatment), high-density lipoprotein cholesterol (HDL) less than 1.3 mmol/L (or on treatment), FPG greater than or equal to 5.6 mmol (or known AGT), and systolic blood pressure (SBP) greater than or equal to 130 mm Hg and/or diastolic blood pressure (DBP) greater than or equal to 85 mm Hg (or on treatment with a history of hypertension) [14].

### Statistical Analyses

Sample size calculation was based on AGT prevalence difference calculated from the HAPO-FUS [13]. Thirty-five participants in each group were required to detect a 32% prevalence difference with an 80% power for a 2-sided type I error rate of 5%. Data was analyzed using SPSS version 21 (IBM). Continuous data were summarized with means ± SD for normally distributed data and medians + minimum to maximum for skewed data. The *t* test was used to determine significant differences in means between 2 groups, and analysis of variance was used to compare the means among 3 groups. Categorical data were summarized with counts and percentages. The Pearson χ^2^ test was used to determine significant differences between groups. Logistic regression analyses were used to compare the prevalence of AGT between groups adjusted for various factors and reported in odds ratio (±95% CI).

## Results

**Figure 1.**
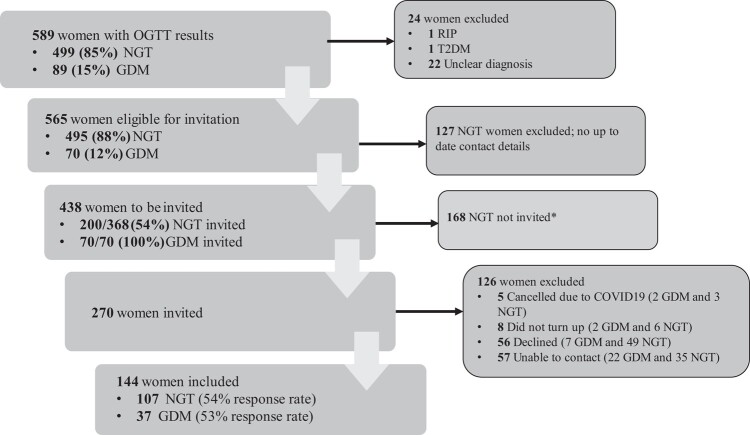
Flowchart of patient recruitment. *Eligible women with NGT at index pregnancy were invited in batches of 100 women selected randomly. Only 2 batches were invited before further recruitment was curtailed by the COVID-19 pandemic. GDM, gestational diabetes mellitus; NGT, normal glucose tolerance; OGTT, oral glucose tolerance test; T2DM, type 2 diabetes mellitus.

Patient recruitment is shown in Fig. 1. A total of 589 women were screened for GDM between June and December 2010. There were 144 participants who attended the follow-up study. Using the WHO 2013 criteria, the cumulative prevalence of GDM during this period was 15.1% (n = 89). Following predefined exclusion criteria, 565 women were eligible for this cohort study, 70 (12.4%) with GDM and 495 (87.6%) with NGT, from whom 200 were selected. A total of 107 women (53.5%) out of 200 invited women with NGT and 37 of 70 (52.9%) with GDM were included in the study.

Baseline characteristics at index pregnancy are shown in Supplementary Table S1 [15]. Although the total overall cohort was largely White, non-White women were more likely to be diagnosed with GDM (4.9% vs 13.5%, NGT vs GDM; *P* < .01). Seven (4.8%) women were non-White. Five of these women had GDM and 2 had NGT. Women with GDM were more likely to have a prior history of GDM (5.6% vs 24.3%, NGT vs GDM; *P* < .01), a higher BMI (27 vs 31, NGT vs GDM; *P* < .01), and higher glucose levels at all time points of the OGTT compared to women with NGT. Index pregnancy outcomes were similar between groups (Supplementary Table S2) [16].

Demographics of women consented to the present cohort are shown in Table 1. Women with prior GDM had a higher BMI (29.4 vs 27.1; *P* = .03), neck circumference (36.7 vs 35.4 cm; *P* = .02), waist-to-hip ratio (0.91 vs 0.86; *P* = .02), and SBP (124 vs 118 mm Hg; *P* = .01). Three women, all with prior GDM, were currently treated with metformin. None of the patients were treated with insulin or other injectable therapy.

**Table 1.** Current baseline characteristics

Outcome	All n = 144	GDM n = 37	NGT n = 107	*P*
Age, y*^a^*	45.7 (4.4)	46.3 (4.5)	45.5 (4.3)	.34
Gravida, n*^b^*	3 (1-15)	4 (1-15)	3 (1-9)	.04
Family history of diabetes, n (%)				
First degree	45 (10.4)	11 (29.7)	34 (31.8)	.83
Second degree	35 (24.3)	8 (21.6)	27 (2.5)	
BMI*^a^*	27.7 (5.6)	29.4 (5.5)	27.1 (3.2)	.03
Neck circumference, cm*^a^*	35.8 (2.9)	36.7 (3.1)	35.4 (2.7)	.02
Waist circumference, cm*^b^*	91.5 (47.0-135.0)	93 (76.5-129.5)	91 (47.0-135.0)	.09
Waist to hip ratio*^b^*	0.87 (0.52-1.08)	0.91 (0.76-1.08)	0.86 (0.52-1.07)	.02
SBP, mm Hg*^a^*	120 (14)	124 (13)	118 (13)	.01
DBP, mm Hg*^a^*	71 (11)	73 (9)	70 (11)	.19
Oral hypoglycemic treatment, n (%)	3 (2.1)	3 (8.1)	0 (0)	<.01
LDL, mmol/L*^b^*	2.7 (1.0-6.0)	2.9 (2.0-5.0)	2.6 (1.0-6.0)	.057
Triglycerides, mmol/L*^b^*	0.9 (0.4-8.1)	1.0 (0.5-2.5)	0.8 (0.4-8.1)	.27
HDL, mmol/L*^b^*	1.6 (0.7-2.9)	1.4 (0.9-2.9)	1.7 (0.7-2.8)	.13
Chol:HDL*^b^*	2.9 (1.8-9.3)	3.2 (1.8-5.9)	2.7 (1.8-9.3)	.054

Abbreviations: BMI, body mass index; Chol, cholesterol; DBP, diastolic blood pressure; GDM, gestational diabetes mellitus; HDL, high-density lipoprotein cholesterol; LDL, low-density lipoprotein cholesterol; NGT, normal glucose tolerance; SBP, systolic blood pressure.

^*a*^Mean (±SD).

^*b*^Median (minimum-maximum).

Parameters of glucose abnormalities are shown in Table 2. The mean FG (5.5 vs 4.9 mmol/L, GDM vs NGT; *P* = .02) and 2-hour PG (6.9 vs 5.2 mmol/L, GDM vs NGT; *P* < .01) were significantly different between women with prior GDM and NGT. Forty-three percent of women (16/37) with GDM in the index pregnancy had AGT at reassessment. Four of these 16 women (25%) had known T2DM. This compared to 4.7% (5/107) of women with prior NGT developing AGT at reassessment with only 1 case (0.9%) of T2DM. Mean HbA_1c_ was significantly higher in GDM vs NGT women (37.4 vs 34.5 mmol/mol; *P* < .01). An additional 4 (10.8%) women with prior GDM and 7 (6.5%) with NGT at index pregnancy would have been classified as AGT if HbA_1c_ was used in the definition of AGT. Of those who developed AGT, more women in the NGT group were in the BMI greater than or equal to 30 category compared to the GDM group (60.0% vs 43.8%, NGT vs GDM; *P* = .04).

**Table 2.** Development of abnormal glucose tolerance at follow-up

Outcome	GDM n = 37	NGT n = 107	*P*
AGT*^a^*, n (%)	16 (43.2)	5 (4.7)	<.01
Prediabetes	12 (32.4)	4 (3.7)	
T2DM	4 (10.8)	1 (0.9)	
Abnormal FPG, n (%)			
Prediabetes	10 (27.0)	2 (1.9)	<.01
T2DM	3 (8.1)	1 (0.9)	
Abnormal PPG, n (%)			
Prediabetes	7 (18.9)	3 (2.8)	<.01
T2DM	3 (8.1)	1 (0.9)	
Known prediabetes, n (%)	3 (8.1)	0 (0)	<.01
Known T2DM, n (%)	2 (5.4)	0 (0)	<.01
Abnormal HbA_1c_, n (%)			
Prediabetes	11 (29.7)	9 (8.4)	<.01
Diabetes	1 (2.7)	1 (0.9)	
OGTT glucose, mmol/L*^b^*			
0 min	5.5 (0.9)	4.9 (1.4)	.02
120 min	6.9 (2.9)	5.2 (2.4)	<.01
HbA_1c_, mmol/mol*^b^*	37.4 (4.3)	34.5 (5.5)	<.01
HOMA-IR*^c^*	2.31 (0.46-20.23)	1.58 (0.38-11.89)	<.01
AGT according to BMI category*^d^*			
<30, n (%)	9 (56.3)	2 (40.0)	.04
≥30, n (%)	7 (43.8)	3 (60.0)	

Abbreviations: AGT, abnormal glucose tolerance; BMI, body mass index; FPG, fasting plasma glucose; GDM, gestational diabetes mellitus; HbA_1c_, glycated hemoglobin A_1c_; HOMA-IR, homeostatic model assessment for insulin resistance; NGT, normal glucose tolerance; OGTT, oral glucose tolerance test; PPG, postprandial plasma glucose; T2DM, type 2 diabetes mellitus.

^*a*^Abnormal glucose tolerance defined using abnormal fasting plasma glucose and/or abnormal PPG only.

^*b*^Mean (±SD).

^*c*^Median (minimum-maximum).

^*d*^For GDM, n = 16 and n = 5 for NGT.

The metabolic risk profiles of the participants are shown in Table 3. When compared to women with current NGT, women with AGT had a significantly poorer metabolic profile including higher BMI, blood pressure, fasting and 2-hour PG, HbA_1c_, and cholesterol-to-HDL ratio. Metabolic syndrome rates were significantly higher in women with AGT compared to NGT (60.0% vs 62.5% vs 10.6%, T2DM vs prediabetes vs NGT; *P* < .01). Insulin resistance as measured by HOMA-IR was highest in those with T2DM compared to those with prediabetes and NGT (8.2 vs 3.3 vs 1.6, T2DM vs prediabetes vs NGT; *P* < .01).

**Table 3.** Metabolic profile of patients with abnormal glucose tolerance at follow-up

Outcome	AGT		NGT	*P*
	N = 21		N = 123	
	Prediabetes N = 16	T2DM N = 5		
BMI*^a^*	29.9 (5.8)	36.0 (7.8)	27.1 (5.0)	<.01
BMI category				
<30, n (%)	10 (62.5)	1(20.0)	95 (77.2)	.01
≥30, n (%)	6 (37.5)	4 (80.0)	28 (23.4)	
Neck circumference, cm*^a^*	36.5 (3.0)	39.0 (4.7)	35.5 (2.7)	.01
Waist to hip ratio*^a^*	0.91 (0.77-1.08)	0.91 (0.84-1.04)	0.86 (0.52-1.07)	.057
Waist circumference, cm*^b^*	94 (76.5-131.0)	113 (84.5-135.0)	91 (47.0-129.0)	<.01
Waist circumference ≥ 88 cm, n (%)	13 (81.3)	4 (80.0)	80 (65.0)	.36
SBP*^a^*	133 (8)	126 (15)	118 (13)	<.01
DBP*^a^*	80 (8)	78 (13)	70 (11)	<.01
Antihypertensive treatment, n (%)	2 (12.5)	1 (20.0)	3 (2.3)	.03
FPG, mmol/L*^a^*	5.7 (0.6)	9.4 (5.1)	4.8 (0.3)	<.01
120-min glucose, mmol/L*^a^*	7.8 (2.0)	15.8 (6.0)	5.0 (1.1)	<.01
HbA1c, mmol/mol*	38.0 (4.1)	50.2 (18.7)	34.3 (3.1)	<.01
HOMA-IR*^b^*	3.3 (1.0-8.8)	8.2 (1.41-21.0)	1.6 (0.4-11.3)	<.01
LDL, mmol/L*^b^*	2.9 (1.7-4.5)	3.3 (2.0-3.8)	2.6 (1.3-5.8)	0.53
Triglycerides, mmol/L*^b^*	1.4 (0.6-2.3)	1.4 (0.7-8.1)	0.8 (0.4-2.5)	<.01
HDL, mmol/L*^b^*	1.5 (0.9-2.3)	1.3 (0.7-1.6)	1.7 (0.9-2.9)	.051
Chol:HDL*^b^*	3.2 (1.9-5.9)	4 (3.2-9.5)	2.8 (1.8-5.6)	<0.01
ALT, IU/L*^a^*	25 (18)	40 (20)	18 (8)	<.01
Metabolic syndrome, n (%)	10 (62.5)	3 (60.0)	13 (10.6)	<.01

Abbreviations: AGT, abnormal glucose tolerance; ALT, alanine transaminase; BMI, body mass index; Chol, cholesterol; DBP, diastolic blood pressure; FPG, fasting plasma glucose; HDL, high-density lipoprotein; HOMA-IR, homeostatic model assessment for insulin resistance; LDL, low-density lipoprotein; NGT, normal glucose tolerance; SBP, systolic blood pressure; T2DM, type 2 diabetes.

^*a*^Mean (±SD).

^*b*^Median (minimum-maximum).

The odds ratio of developing AGT 10 years after a diagnosis of GDM was 15.5 (95% CI, 5.1-47.1). This remained significant after correcting for BMI, ethnicity, family history, parity, and GDM before or after index pregnancy. Similarly, the odds ratio of developing metabolic syndrome was higher in those with AGT, 6.67 (95% CI, 2.75-16.17; *P* < .01), and remained significant after correcting for similar potentially confounding factors.

Breastfeeding and insulin treatment at index pregnancy did not have any effect on the development of AGT in this cohort. None of the participants had a known history CVD and independent assessment of their ECGs showed no abnormalities.

## Discussion

As far as we are aware, this is the first long-term follow-up study demonstrating metabolic outcomes of a group of women with GDM from routine clinical practice diagnosed and treated using the WHO 2013 criteria. Our study demonstrates that the risk of AGT remains high despite GDM treatment during pregnancy. In our study, the prevalence of AGT at 10 years after an index pregnancy affected by GDM was only slightly lower than that documented in the HAPO-FUS (43% vs 52%) [13]. Of note, in the HAPO-FUS women with NGT had a 20% prevalence of developing AGT compared to 4.7% in our study. This does not seem to be explained by BMI as participants in our study had a similar BMI to those in the HAPO-FUS. A prior study by our group showed a 5-year cumulative prevalence of AGT after GDM of 26% [17]. Our study shows that after 10 years, the risk of AGT remains significant despite treatment during pregnancy and thus these women need life-long follow-up. This is also supported by a recent study showing a linear increase in the development of T2DM after more than 20 years of follow-up [18].

Treatment of GDM diagnosed using the much lower cutoff points of the WHO 2013 criteria confers improved perinatal outcomes [19, 20] but does not seem to confer metabolic memory and protection from AGT and adverse CVD risk factors in later life. In our study, women treated with insulin were as likely as women on medical nutritional therapy to develop AGT. Some studies using varying GDM diagnostic criteria have shown increased risk of developing AGT with insulin treatment during pregnancy compared to treatment with medical nutritional therapy [21, 22]. We had anticipated that with the relatively lower cutoff points used to diagnose GDM in our study, women treated with insulin may have lower rates of AGT. In our center insulin is used as first-line treatment for GDM, thus none of our patients were treated with metformin during pregnancy. It therefore remains to be seen whether treatment with metformin during pregnancy alone or in addition to insulin attenuates the risk of developing AGT and metabolic syndrome in the future. In women with polycystic ovary syndrome, metformin seems to be similar to placebo in terms of long-term metabolic outcomes including AGT [23]. A randomized controlled trial of metformin vs placebo in the treatment of GDM has recently been published, and long-term data from this trial may be able to shed light on whether metformin use during pregnancy has any effect on the long-term metabolic health of the woman [24]. Another open-label randomized controlled trial aims to assess long-term use of metformin administered up to 1 year after delivery in women with GDM [25].

The diabetes prevention program showed a reduction in the risk of developing diabetes in a high-risk cohort of patients (including women with prior GDM) with prediabetes and obesity [26]. In the diabetes prevention program study, patients randomly assigned to lifestyle interventions had more weight loss compared to placebo and metformin. In our study, 80% of obese patients developed T2DM compared to 20% with BMI less than 30. In general, participants lost weight in the GDM group over the 10 years (BMI 31.0 [±5.2] at index pregnancy to 29.4 [±5.5] at follow-up). In addition, patients with a BMI of 30 or greater were more likely to develop AGT independent of GDM status at index pregnancy. Thus, weight loss remains essential in diabetes prevention after GDM. Although others have shown that breastfeeding reduces the risk of diabetes in the long term in women with prior GDM [27], we did not find similar results in this study.

CVD risk factors were significantly increased in women with AGT compared to those with NGT, as shown in Table 3. In particular, women had an increased risk of metabolic syndrome, insulin resistance, obesity, and clinically evident hypertension (need for antihypertensive treatment). Metabolic syndrome has been shown to increase the risk of T2DM and CVD [28]. In addition, insulin resistance has been shown to increase the risk of T2DM [28]. Obesity, another independent risk factor for CVD and mortality [29], was significantly higher in women who developed T2DM in our cohort, underpinning the importance of weight loss as a key factor in the long-term health of this high-risk group. Although there were no documented CV events in our study, others have shown increased risk in women with prior GDM [3, 30, 31]. Two of these studies assessed CVD events in an older population compared to those in our study. Lee et al used UK Biobank data of women in their 50s [31] compared to those in their 40s in this study. In the Danish study by Yu et al, women were followed up for a median of 16 years compared to our study's 10 years [3]. However, despite women not developing any evident CVD in this study, they still remain at increased risk as shown by their poor metabolic profile at follow-up. Therefore, modifiable risk factors such as obesity, hypertension, insulin resistance, hyperglycemia, and dyslipidemia should be aggressively targeted soon after childbirth.

Although 4 (10.8%) women with GDM and 7 (6.5%) women with NGT at index pregnancy had a normal OGTT at 10-year follow-up, their HbA_1c_ was in the prediabetes range. This discordance is similar to other studies assessing risk of prediabetes and T2DM in at-risk groups [32, 33]. Although some studies have suggested discordance in HbA_1c_ due to ethnicity [34], our study did not support this finding. In addition, this discordance was not explained by age, family history of diabetes, or parity. Some advocate screening for diabetes with a fasting glucose and/or HbA_1c_ for simplicity; however, in our cohort 5 (out of 21) women had IGT (prediabetes range) with a normal FG. Three of these women, however, had an abnormal HbA_1c_ (prediabetes range), therefore if combined fasting and HbA_1c_ were used for screening, 2 (9.5%) women would have been missed. This is important because the evidence supports interventions for primary prevention of T2DM in individuals with IGT compared to isolated impaired FPG or prediabetes defined by HbA_1c_ criteria [35].

This study has some limitations. First, only half of the patients invited in each group participated in the study. The study was halted earlier than had been anticipated due to the COVID pandemic, thus there were relatively small numbers of participants in this study. A study with a much larger sample size may be needed to see an effect on outcomes such as CVD. Second, CVD was screened with ECG and patient recall. All participants were asymptomatic, and current guidance does not recommend ECG as a screening tool for ischemic heart disease [36]. Perhaps cardiac biomarkers such as N-terminal pro-brain natriuretic peptide, troponin, and C-reactive protein, which have been shown to predict CVD in asymptomatic individuals without a prior CVD history, could more reliably identify high-risk patients in future studies [37, 38]. Last, AGT was based on single abnormal OGTT results in asymptomatic (albeit high-risk) patients. Current guidance would recommend repeat testing in asymptomatic patients [39].

In conclusion, this study shows that despite treatment during pregnancy, women with GDM diagnosed using the WHO 2013 criteria remain at increased risk of AGT and adverse metabolic profile 10 years after the index pregnancy. Continued effort to minimize CVD risk and prevent noncommunicable disease in this group is warranted.

## Data Availability

Data generated during this study are not publicly available but are available from the corresponding author on reasonable request.

## Abbreviations

AGT: abnormal glucose tolerance
BMI: body mass index
CVD: cardiovascular disease
DBP: diastolic blood pressure
ECG: electrocardiogram
FG: fasting glucose
FPG: fasting plasma glucose
HAPO: Hyperglycemia and Adverse Pregnancy Outcome
HAPO-FUS: HAPO follow-up study
HbA_1c_: glycated hemoglobin A_1c_
HDL: high-density lipoprotein
HOMA-IR: homeostatic model assessment for insulin resistance
IADPSG: International Association of Diabetes and Pregnancy Study Groups
IFG: impaired fasting glucose
IGT: impaired glucose tolerance
LDL: low-density lipoprotein
NGT: normal glucose tolerance
OGTT: oral glucose tolerance test
PG: plasma glucose
SBP: systolic blood pressure
T2DM: type 2 diabetes mellitus
WHO: World Health Organization
